# Assessment of Spanish Food Consumption Patterns during COVID-19 Home Confinement

**DOI:** 10.3390/nu13114122

**Published:** 2021-11-17

**Authors:** Ana Maestre, Isabel Sospedra, José Miguel Martínez-Sanz, Ana Gutierrez-Hervas, José Fernández-Saez, José Antonio Hurtado-Sánchez, Aurora Norte

**Affiliations:** 1 Faculty of Health Sciences, University of Alicante, 03690 Alicante, Spain; anamaestreciru@gmail.com; 2 Research Group on Food and Nutrition (ALINUT), Nursing Department, Faculty of Health Sciences, University of Alicante, 03690 Alicante, Spain; isospedra@ua.es (I.S.); josemiguel.ms@ua.es (J.M.M.-S.); ja.hurtado@ua.es (J.A.H.-S.); auora.norte@ua.es (A.N.); 3 Nursing Department, Faculty of Health Sciences, University of Alicante, 03690 Alicante, Spain; 4 Unitat de Suport a la Recerca Terres de l’Ebre, Fundació Institut Universitari per a la Recerca a l’Atenció Primària de Salut Jordi Gol i Gurina (IDIAPJGol), 43500 Tortosa, Spain; j.fernandez@ua.es; 5 Unitat de Recerca, Gerència Territorial Terres de l’Ebre, Institut Catalá de la Salut, 43500 Tortotsa, Spain; 6 Facultat de Enfermería, Campus Terres de l’Ebre, Universitat Rovira i Virgili, 43003 Tortosa, Spain

**Keywords:** COVID-19, home confinement, food consumption, dietary pattern

## Abstract

People’s eating habits and lifestyle can have a negative impact on health. In situations of difficulty or socioeconomic crisis, these habits tend to be modified, leading to unhealthy dietary patterns that result in an increase of chronic non-communicable diseases (NCDs). Previous studies have indicated that, due to the state of alarm imposed in Spain to combat the spread of COVID-19, an increase in the purchase of non-core products occurred, along with a decrease in the daily physical activity of the population. This could be a risk factor for COVID-19 infection. The objective of this observational study was to analyze the dietary pattern of the Spanish population during home confinement and to compare it with the pattern of habitual consumption collected in the last National Health Survey, analyzing the possible changes. More than half of the respondents in the sample increased their consumption of sweets and snacks during confinement, while the consumption of fresh products decreased. Most claimed to be emotionally hungry, leading to an increase in their daily energy intake. The stress and anxiety generated by confinement could be the cause of the increased consumption of products rich in sugars and saturated fats, which are associated with greater stress and anxiety.

## 1. Introduction

In Spain, as in many other countries during the year 2020, a state of alarm was declared due to the situation generated by the COVID-19 disease, related to SARS-CoV-2. The transmission of this infectious disease is very high due to its incubation period [1,2]. In order to slow the spread of the virus and to be able to cope with the hospital saturation generated by the high number of infected patients [3,4], the state of alarm included mobility restriction and confinement measures. These measures led to changes in the lifestyle of the population, including their eating habits. The state of alarm also had socioeconomic impacts due to the paralysis of the work of non-essential professions, which could have aggravated the state of health of the population due to a loss of income and thus of food purchase power [5]. Recent studies show that, in a situation of economic crisis, there is a greater likelihood of adhering to poor eating habits, which can have a negative impact on the population [6,7]; thus, the restrictions imposed could have repercussions for the health status of the Spanish population.

After a state of alarm was declared throughout Spain, there were many reports of changes in the most purchased food. Initially, there were massive purchases of basic products, and the acquisition of fresh products remained stable [8,9,10]. However, as the period of home confinement was extended, the purchase of products essential for a healthy diet, such as oil, rice, pulses, and pasta, declined [11]. Furthermore, there was an increase in the purchase of non-essential and unhealthy foods, such as beer, chips, olives, and chocolate bars. There was also a massive purchase of flour and yeast for the production of homemade pastry products, as the population looked for new pastimes during the confinement, especially if living with children [12].

If we add to all the above a decrease in regular physical activity due to the restriction of mobility, it is to be expected that, in general, the population would have reduced its energy expenditure and increased its daily energy consumption through the ingestion of non-basic products, such as homemade pastries (Chen et al., 2020). This would produce an increase in the risk of being overweight or obese, and these pathologies increase the probability of complications in COVID-19 infections [13,14,15]. Thus, this population group had a higher risk of complications after COVID-19 infection, as is the case with the population over 60 years of age and pregnant women [16,17].

It is important to point out that if negative changes in the food consumption pattern occur, such as an increase in the consumption of alcohol, chocolate, pastries, or snacks, as well as a decrease in energy expenditure, this situation can cause people to become a risk group for COVID-19 infection [18,19]. Scientific evidence shows that a healthy diet is essential to preserve the physical, psychological, and social health of the population and in order to prevent and treat chronic non-communicable diseases (NCDs) [11,20,21]. Due to these factors, there is a need to know, analyze, and quantify the changes produced in the dietary pattern of the Spanish population during the period of confinement resulting from the pandemic, as has been done in some other countries [22,23]. This will assist the formulation of new recommendations intended to reduce the possible health risks caused by such situations. Therefore, the objectives of this study were to analyze the dietary pattern of the Spanish population during home confinement, to compare it with the pattern of habitual consumption found in the last National Health Survey, and to analyze the changes.

## 2. Materials and Methods

The study had a cross-sectional observational design. The population under study comprised persons over 18 years of age residing in Spain during the period of home confinement caused by the COVID-19 outbreak in March 2020. The sample was selected by convenience sampling and consisted of 1640 people, 1142 women and 498 men. Participants were informed about the objective of the study, and they were given a signed written informed consent. The study was conducted according to the guidelines of the Declaration of Helsinki, and it was approved by the University of Alicante Research Ethics Committee (permit no. #UA-2021-00 02).

The variables of the study were collected through an online self-report questionnaire. The distribution of the questionnaire began on 1 April 2020, and data were collected until the last day of home confinement on 4 May 2020. The questionnaire collected sociodemographic variables (age, sex, and employment status (students, retirees, unemployed or temporarily unemployed people, people teleworking, people working, people on temporary sick leave or on holiday, and homemakers)) in addition to variables related to the population’s diet, classified into food groups. The same food consumption frequency questionnaire (FFQ) used by the Spanish SSS was used to collect food information. This FFQ consists of 18 questions on the frequency of consumption of foods and beverages classified as fruit; meat; fish; eggs; bread and cereals; pasta, rice, and potatoes; salads and vegetables; pulses; cured meat products and cold cuts; dairy products; sweets; sugary sodas; fast food; savory snacks; and natural fruit juice. The results of this FFQ were used to compare the results with the results obtained in the latest National Health Survey (NHS) [24] and with the food consumption recommendations of the Spanish Agency for Food Safety and Nutrition (AESAN) and the World Health Organization (WHO) [25,26]. In addition to information on frequency of food consumption, information on alcohol consumption and emotional hunger was also collected.

The questionnaire was created through the Google questionnaires platform and was distributed mainly using WhatsApp or email. Once the questionnaire response period was closed, a database with Excel software was created. A descriptive analysis of the sample, based on frequency and percentage, was performed for categorical variables. The mean, standard deviation, and median were used for continuous variables. To determine whether there were statistically significant differences between the food consumption during confinement and the usual consumption habits of the population, a ratio difference contrast was used to compare the food consumption frequency variables for the data collected during confinement with those of the data obtained in the last published Spanish SSN.

The statistical analyses of the results were carried out with the statistical package SPSS 20.0.

The study design met the STROBE-nut statements according to Lachat et al. 2016 [27].

## 3. Results

The mean age of the participants was 38 ± 12 years, while 98.4% of the sample was below 65 years of age. Table 1 shows the employment situation during the confinement period; it can be highlighted that more than 36% of the population were teleworking during this period, almost 23% were subject to an ERTE (temporary labor force adjustment plan), and less than 20% of the population were going to work as normal. According to these data, more than 80% of the respondents were completely confined at home.

Based on the food consumption questionnaire responses, more than 70% of the study population consumed greater amounts of non-essential foods, such as sweets and cured meat products; more than half had a higher than recommended consumption of meat; and approximately 60% failed to comply with the recommended frequency of consumption of fish, by a wide margin. However, more than half of the sample reached the recommended consumption of fruit, vegetables, and dairy products (see Table 2). However, when comparing the consumption before and during the confinement, Table 3 shows that the consumption of fresh fruit, fish, cured meat products, cold cuts, and sugary sodas generally decreased during confinement. Conversely, the consumption of meat, eggs, dairy products, pulses, potatoes, cereals, and their derivatives increased. However, the most striking thing is that more than 28% of the respondents declared that their consumption of snacks and sweets increased during this period.

In addition, more than 40% of the respondents reported an increase in the total amount of food eaten during confinement, and they said they were emotionally hungry or anxious, which they “managed” through their food habits. Of the respondents disclosing these variations in consumption, a majority were women. With regard to alcoholic drinks, almost 20% of the sample increased their consumption, and in this case, men were in the majority. The alcoholic drinks consumed most often during confinement were wine and beer.

When comparing the consumption data published in the last NHS with the data collected during confinement, by means of a contrast of proportions, a significant increase was found in almost all categories of meat and egg consumption frequencies during confinement. However, the consumption of fish and pulses, as well as bread and cereals, decreased. Regarding pasta, rice, and potatoes, the frequency of consumption increased to 4–6 times per week, while the daily consumption of vegetables increased slightly. The frequency of the consumption of cured meat products and cold meats, as well as dairy products, decreased to 3 to 6 times per week (see Table 4). It should be noted that sweets consumption changed from occasional to 4 to 6 times a week; similarly, the frequency of consumption of snacks increased from weekly to daily. The intake of sugary sodas decreased significantly.

## 4. Discussion

The aims of this study were to identify and evaluate the main changes in the eating habits of the Spanish population during the home confinement imposed during the COVID-19 state of alarm and to compare these data with those of the last NHS carried out in Spain in 2017.

The main results indicate that almost 50 per cent of the respondents increased the amount of food consumed and that they also suffered from emotional hunger and anxiety. These data coincide with those of a recent study, which concluded that the COVID-19 pandemic has been characterized by an increase in psychological disorders, such as anxiety, stress, and depression, in the general population and, more specifically, in university students [28]. In addition, according to Lee et al. in 2016 [29], there is a higher prevalence of these disorders in women and in people between 20 and 40 years old. Thus, it would be relevant to take this aspect into account, since almost 70% of the respondents of this study were women, and the overall average age was between those ages. Another study characterized stress and anxiety as causes of high consumption of foods rich in saturated fat and added sugars, which are associated with increased anxiety [30]. That is, the stress generated by home confinement can lead to high consumption of such products, which are associated with greater anxiety and stress, which can also be increased by sedentary behavior [31].

In our work, there was a notable increase in the consumption of products such as meat, eggs, pasta, rice, and potatoes and a smaller increase in vegetable consumption, while for fruit, dairy products, fish, and pulses, the consumption decreased. Excessive consumption of the former foods can cause an overeating that, together with the low level of physical activity that was common during confinement due to mobility restrictions, could lead to important metabolic changes in the body, such as NCDs; these may be harmful to the immune system and may contribute to a higher risk of contracting the COVID-19 disease [32]. Another study found that simply reducing daily physical activity can affect glycemic control in healthy individuals, without associated pathologies, due to an increase in postprandial glucose [33]. Therefore, if a change in food consumption with an increase in energy intake is added to this, the metabolic consequences would include insulin resistance, weight gain due to excess energy, and changes in the body composition of the individual, with the corresponding repercussions such as obesity, cardiovascular diseases, and type 2 diabetes mellitus [32,34].

In addition, our results show that the consumption of fruit, fish, and vegetables declined, and the values were not in line with EFSA recommendations, whereas the values of the pre-confinement period were. According to Ulrich-Lai et al. in 2015 [35], in situations of stress and social anxiety, there is a tendency to choose more palatable and less healthy foods, while in situations of chronic stress, the daily energy intake is usually reduced by a decrease in appetite. Hence, the most significant increase observed in this period was for sweets and snacks, since more than half of the respondents stated that they consumed sugary products, considered appropriate only for occasional consumption [25], on a daily basis. However, the intake of these most appetizing foods to reduce stress and anxiety can produce the opposite effect, depending on the composition of the food and the system of stress regulation, because a protein-rich, carbohydrate-poor diet generates a longer-term release of brain serotonin than a high-carbohydrate, low-protein diet. Thus, the former diet generates a sense of pleasure, which is what the population seeks when consuming the least healthy foods, and it serves as a reinforcement to face stress and anxiety [36].

Based on all this, we could assume that there was a worsening of the dietary patterns of the population due to the increase in anxiety produced by confinement that resulted in an increase in the consumption of these products, since, in both the data self-reported by the participants and the contrast of proportions, there was an increase in snacks and in products rich in sugars, along with a decrease in fresh products. Masana et al. in 2019 [37] observed that diets rich in sugars, fats, processed foods, and meat contribute to an increase in anxiety through an increase in inflammatory cytokines and corticosterone. Healthy diets with a high consumption of fruit and vegetables can contribute in a beneficial way to a decrease in anxiety and stress. Thus, healthier dietary patterns should be promoted, not only to lessen stress and anxiety, but also because foods rich in antioxidants, such as vegetables, help to improve the immune system and thus inhibit the development of diseases such as COVID-19 [38,39]. Recent studies have shown that diet and individual nutrients can influence systemic markers of immune function and inflammation. Moreover, the nutritional status could be used as indicator to assess the immune status of patients with COVID-19 [40,41]. It is also important to take into account the energy balance in any diet, since the prevalence of obesity is increasing worldwide, and there is a need to act to curb it [39]. Therefore, a lower weight gain or a healthy weight loss should be the aim for each individual who is overweight or obese, and this needs to be maintained in the long-term to minimize this disease globally. The achievement of a negative daily energy balance through daily physical activity would produce an optimal state of health in the population, in addition to being an effective treatment for NCDs, as observed by Jiménez-Pavón et al. in 2020 [42]. In this study, it can be observed how any type of physical activity would lead to an improvement in the state of health; depending on the situation, the frequency and intensity of the exercise could be adapted. In this way, obesity would be reduced worldwide, decreasing the mortality risk of COVID-19 [43]. Even so, if a healthy and balanced diet limited in added sugars and saturated fats is not followed, changes in physical activity would not have the same impact on the population [44].

One of the main limitations of this study was the telematic sampling system. To ensure an adequate number of responses, weekly reminders were sent by the same means of dissemination as the questionnaire. In addition to maximizing the data collection, special attention was paid to the format details of the questionnaire, to achieve a short, simple, and pleasant design and content. Since these data were self-reported by the participants, they contain several potential sources of bias, because they could rarely be independently verified. One of these biases is related to weight and height data and, consequently, the BMI values, because self-reported data could lead to an understatement of BMI values, as has been described before [45]. On the other hand, in self-reported food consumption surveys the eating frequencies and serving sizes specified may not represent the respondent’s usual intake. Moreover, it requires a certain level of literacy and cognitive skills. Thus, it could lead to an over- or under-estimation of the data collected [46]. Another possible limitation is the fact that more than 65% of the sample belonged to the Valencian Community. The consumption of some products could vary in other areas of Spain, according to their availability and local customs.

To conclude, we can state that there were changes in food consumption during the home confinement that occurred during the COVID-19 pandemic. These changes generally included an increase in the amount of food ingested, leading to an increase in daily energy intake, coupled with an increase in the frequency of consumption of sweet foods and snacks and a decrease in the consumption of fruit, vegetables, fish, and pulses. Together, these could contribute to an increased risk of COVID-19 infection. For similar situations in which physical activity is decreased, it would be advisable to reduce the daily energy intake and to have a healthy and balanced diet based on the Mediterranean diet (greater content of fruits and vegetables), with healthy fats and foods rich in protein: vegetables (such as pulses), meat, fish, and eggs. By prioritizing these foods, the consumption of sugary, high-fat products rich in trans- and saturated fats, such as sweets and snacks, is limited. This can help to prevent NCDs such as obesity, diabetes mellitus, and cardiovascular diseases in the population; these result in a worse prognosis with regard to overcoming the COVID-19 disease that is so prevalent around the world at this time.

## Figures and Tables

**Table 1:** Description of the employment situation of the Spanish population during the COVID-19 pandemic confinement, for the total sample and by sex.

Employment Situation	Men *n* (%)	Women *n* (%)	Total *n* (%)
Students	56 (11.2)	175 (15.3)	231 (14.1)
Retirees	23 (4.6)	16 (1.4)	39 (2.4)
Teleworking	184 (36.9)	414 (36.3)	598 (36.5)
Unemployed or temporarily unemployed (ERTE)	96 (19.2)	279 (24.4)	375 (22.9)
Working	117 (23.5)	172 (15.1)	289 (17.6)
Temporary sick leave or holidays	21 (4.2)	72 (6.4)	93 (5.7)
Homemakers	1 (0.2)	14 (1.2)	15 (0.9)

**Table 2:** Food consumption frequency during the period of confinement imposed during the COVID-19 pandemic and the percentage suitabilities with respect to the consumption recommended by the Spanish Society of Community Nutrition (SENC).

Food Groups	Mean Consumption Frequency					
	Never *n* (%)	Less than 1 Time per Week *n* (%)	1 or 2 Times per Week *n* (%)	3 Times per Week *n* (%)	4–6 Times per Week *n* (%)	1 Time or More per Day *n* (%)
Fruit	35 (2.1)	56 (3.4)	91 (5.5)	153 (9.3)	223 (13.6)	1082 (66.0) *
Meat	69 (4.2)	37 (2.3)	232 (14.1)	472 (28.8) *	612 (37.3)	218 (13.3)
Eggs	19 (1.2)	51 (3.1)	511 (31.2)	598 (36.5) *	356 (21.7)	105 (6.4)
Fish	80 (4.9)	162 (9.9)	746 (45.5)	466 (28.4) *	160 (9.8)	26 (1.6)
Pasta, rice, and potatoes	12 (0.7)	40 (2.4)	385 (23.5)	558 (34.0)	525 (32) *	120 (7.3)
Bread and cereals	16 (1.0)	29 (1.8)	112 (6.8)	147 (9.0)	384 (23.4) *	952 (58)
Vegetables	12 (0.7)	20 (1.2)	78 (4.8)	170 (10.4)	404 (24.6)	956 (58.3) *
Pulses	48 (2.9)	174 (10.6)	843 (51.4)	405 (24.7) *	130 (7.9)	40 (2.4)
Cured and cold meat	183 (11.2)	279 (17.0) *	451 (27.5)	314 (19.1)	246 (15.0)	167 (10.2)
Dairy products	36 (2.2)	30 (1.8)	79 (4.8)	126 (7.7)	289 (17.6)	1080 (65.9) *
Sweets and candy	151 (9.2)	284 (17.3) *	363 (22.1)	227 (13.8)	290 (17.7)	325 (19.8)
Sugary sodas	1091 (66.5)	225 (13.7) *	180 (11.0)	51 (3.1)	49 (3.0)	44 (2.7)
Fast food	556 (33.9)	520 (31.7) *	415 (25.3)	107 (6.5)	29 (1.8)	13 (0.8)
Snacks	377 (23.0)	394 (24.0) *	461 (28.1)	213 (13)	136 (8.3)	59 (3.6)
Natural juice	769 (46.9)	239 (14.6)	255 (15.5)	124 (7.6)	90 (5.5)	163 (9.9)

* *p* < 0.05.

**Table 3:** Comparison of the consumption of food groups by the Spanish population before and during the confinement imposed during the COVID-19 pandemic.

Food Groups	Consumption Less Frequent than before Confinement *n* (%)	Consumption at the Same Frequency as before Confinement *n* (%)	Consumption More Frequent than before Confinement *n* (%)
Fruit	277 (16.9)	1137 (69.3)	226 (13.8)
Meat	119 (7.3)	1342 (81.8)	179 (10.9)
Eggs	74 (4.5)	1312 (80)	254 (15.5)
Fish	370 (22.6)	1109 (67.6)	161 (9.8)
Pasta, rice, and potatoes	168 (10.2)	1177 (71.8)	295 (18)
Bread and cereals	162 (9.9)	1171 (71.4)	307 (18.7)
Vegetables	163 (9.9)	1256 (76.6)	221 (13.5)
Pulses	181 (11)	1243 (75.8)	216 (13.2)
Cured and cold meat	259 (15.8)	1162 (70.9)	219 (13.4)
Dairy products	75 (4.6)	1328 (81)	237 (14.5)
Sweets and candy	255 (15.5)	849 (51.8)	536 (32.7)
Sugary sodas	286 (17.4)	1257 (76.6)	97 (5.9)
Snacks	290 (17.7)	878 (53.5)	472 (28.8)
Natural juice	179 (10.9)	1314 (80.1)	147 (9)

**Table 4:** Contrast of proportions of the consumption frequencies during confinement and according to the last Spanish national health survey.

Food Groups	Consumption Frequency	COVID *n* (%)	Spanish National Health Survey *n* (%)	*p* Value
Fruit	Never	35 (2.1)	478 (2.1)	0.861
	<1 time/week	56 (3.4)	743 (3.2)	0.663
	1–2 times/week	91 (5.5)	1720 (7.4)	0.004
	3 times/week	153 (9.3)	1910 (8.3)	0.135
	4–6 times/week	223 (13.6)	2916 (12.6)	0.255
	>1 time/day	1082 (66)	15,308 (66.3)	0.788
Meat	Never	69 (4.2)	288 (1.2)	<0.001
	<1 time/week	37 (2.3)	558 (2.4)	0.682
	1–2 times/week	232 (14.1)	6642 (28.8)	<0.001
	3 times/week	472 (28.8)	8382 (36.3)	<0.001
	4–6 times/week	612 (37.3)	5370 (23.3)	<0.001
	>1 time/day	218 (13.3)	1827 (7.9)	<0.001
Eggs	Never	19 (1.2)	246 (1.1)	0.723
	<1 time/week	51 (3.1)	1868 (8.1)	<0.001
	1–2 times/week	511 (31.2)	13,475 (58.4)	<0.001
	3 times/week	598 (36.5)	5616 (24.3)	<0.001
	4–6 times/week	356 (21.7)	1556 (6.7)	<0.001
	>1 time/day	105 (6.4)	311 (1.3)	<0.001
Fish	Never	80 (4.9)	518 (2.2)	<0.001
	<1 time/week	162 (9.9)	1908 (8.3)	0.023
	1–2 times/week	746 (45.5)	11,395 (49.4)	0.002
	3 times/week	466 (28.4)	6799 (29.4)	0.375
	4–6 times/week	160 (9.8)	2201 (9.5)	0.766
	>1 time/day	26 (1.6)	250 (1.1)	0.061
Pasta, rice, and potatoes	Never	12 (0.7)	100 (0.4)	0.082
	<1 time/week	40 (2.4)	431 (1.9)	0.101
	1–2 times/week	385 (23.5)	5935 (25.7)	0.046
	3 times/week	558 (34)	8762 (37.9)	0.002
	4–6 times/week	525 (32)	4673 (20.2)	<0.001
	>1 time/day	120 (7.3)	2170 (9.4)	0.005
Bread and cereals	Never	16 (1.0)	376 (1.6)	0.041
	<1 time/week	29 (1.8)	333 (1.4)	0.288
	1–2 times/week	112 (6.8)	709 (3.1)	<0.001
	3 times/week	147 (9)	766 (3.3)	<0.001
	4–6 times/week	384 (23.4)	1089 (4.7)	<0.001
	>1 time/day	952 (58)	19,801 (85.8)	<0.001
Vegetables and salads	Never	12 (0.07)	199 (0.09)	0.580
	<1 time/week	20 (1.2)	403 (1.7)	0.112
	1–2 times/week	78 (4.8)	2159 (9.4)	<0.001
	3 times/week	170 (10.4)	4194 (18.2)	<0.001
	4–6 times/week	404 (24.6)	6594 (28.6)	0.001
	>1 time/day	956 (58.3)	9521 (41.2)	<0.001
Pulses	Never	48 (2.9)	358 (1.6)	<0.001
	<1 time/week	174 (10.6)	2267 (9.8)	0.299
	1–2 times/week	843 (51.4)	14,419 (62.4)	<0.001
	3 times/week	45 (24.7)	4945 (21.4)	0.002
	4–6 times/week	130 (7.9)	2767 (12.0)	<0.001
	>1 time/day	40 (2.4)	2755 (11.9)	<0001
Cured and cold meats	Never	183 (11.2)	1893 (8.2)	<0.001
	<1 time/week	279 (17)	3950 (17.1)	0.921
	1–2 times/week	451 (27.5)	7196 (31.2)	0.002
	3 times/week	314 (19.1)	4495 (19.5)	0.750
	4–6 times/week	246 (15)	2767 (12)	<0.001
	>1 time/day	167 (10.2)	2755 (11.9)	0.034
Dairy products	Never	36 (2.2)	542 (2.3)	0.693
	<1 time/week	30 (1.8)	434 (1.9)	0.884
	1–2 times/week	79 (4.8)	818 (3.5)	0.008
	3 times/week	126 (7.7)	810 (3.5)	<0.001
	4–6 times/week	289 (17.6)	1214 (5.3)	<0.001
	>1 time/day	1080 (65.9)	19,250 (83.4)	<0.001
Sweets	Never	151 (9.2)	2677 (11.6)	0.003
	<1 time/week	284 (17.3)	4084 (17.7)	0.703
	1–2 times/week	363 (22.1)	5251 (22.7)	0.570
	3 times/week	227 (13.8)	2923 (12.7)	0.165
	4–6 times/week	290 (17.7)	2032 (8.8)	<0.001
	>1 time/day	325 (19.8)	6096 (26.4)	<0.001
Sugary sodas	Never	1091 (66.5)	10,656 (46.2)	<0.001
	<1 time/week	225 (13.7)	5265 (22.8)	<0.001
	1–2 times/week	180 (11)	3425 (14.8)	<0.001
	3 times/week	51 (3.1)	1157 (5)	0.001
	4–6 times/week	49 (3)	754 (3.3)	0.540
	>1 time/day	44 (2.7)	1799 (7.8)	<0.001
Fast food	Never	556 (33.9)	8645 (37.4)	0.004
	<1 time/week	520 (31.7)	7463 (32.3)	0.607
	1–2 times/week	415 (25.3)	5558 (24.1)	0.260
	3 times/week	107 (6.5)	818 (3.5)	<0.001
	4–6 times/week	29 (1.8)	316 (1.4)	0.182
	>1 time/day	13 (0.8)	256 (1.1)	0.233
Snacks	Never	377 (23)	8245 (35.7)	<0.001
	<1 time/week	394 (24.0)	7810 (33.8)	<0.001
	1–2 times/week	461 (28.1)	5297 (22.9)	<0.001
	3 times/week	213 (13)	1029 (4.5)	<0.001
	4–6 times/week	136 (8.3)	399 (1.7)	<0.001
	>1 time/day	59 (3.6)	275 (1.2)	<0.001
Natural juice	Never	769 (46.9)	9412 (40.8)	<0.001
	<1 time/week	239 (14.6)	4440 (19.2)	<0.001
	1–2 times/week	255 (15.5)	3491 (15.1)	0.640
	3 times/week	124 (7.6)	1971 (8.5)	0.170
	4–6 times/week	90 (5.5)	1235 (5.3)	0.809
	>1 time/day	163 (9.9)	2506 (10.9)	0.249

## Data Availability

The data presented in this study are available in the tables of this article. The data presented in this study are available on request from the corresponding author.
